# A comprehensive review of the pathogenic mechanisms of *Pseudomonas aeruginosa*: synergistic effects of virulence factors, quorum sensing, and biofilm formation

**DOI:** 10.3389/fmicb.2025.1619626

**Published:** 2025-07-21

**Authors:** Xindan Zhang, Duo Zhang, Di Zhou, Shuai Zheng, Shuang Li, Qinlong Hou, Gen Li, Huiming Han

**Affiliations:** ^1^The School of Basic Medicine, Beihua University, Jilin, China; ^2^The Center for Infection and Immunity, Beihua University, Jilin, China; ^3^Department of Science and Education, Affiliated Hospital of Beihua University, Jilin, China

**Keywords:** *Pseudomonas aeruginosa*, virulence factors, quorum sensing, biofilm, immune evasion

## Abstract

*Pseudomonas aeruginosa* (*P. aeruginosa*) is a ubiquitous opportunistic pathogen and a major cause of nosocomial infections worldwide. It can provoke a spectrum of clinical manifestations-ranging from postoperative wound infections, pressure ulcers, abscesses, and otitis media to life-threatening bacteremia and sepsis, especially in burn patients. Over the past decade, extensive research has elucidated its complex virulence repertoire, including exotoxins, proteases, and siderophores; the hierarchical Quorum Sensing (QS) networks; and its robust capacity for biofilm formation. In this review, we consolidate significant studies published since 2015 to develop a comprehensive framework elucidating the virulence mechanisms of *P. aeruginosa*. Beyond cataloging individual factors, we highlight how QS regulators coordinate toxin production and biofilm maturation, and how these processes converge to facilitate immune evasion. We further examine cross-talk between QS circuits (Las, Rhl, and Pqs), their response to environmental cues, and the modulatory role of host signals. Despite these advances, significant gaps remain: the spatiotemporal interplay among different virulence modules; the precise molecular triggers of biofilm dispersal; and the dynamics of bacterial–host immune interactions *in vivo*. Notably, targeting QS with small-molecule inhibitors has shown promise in attenuating pathogenicity, yet translating these findings into clinical therapies requires more nuanced understanding of resistance emergence and host microbiome effects. We propose that future investigations prioritize (1) the structural biology of QS receptors to guide rational inhibitor design; (2) single-cell and organ-on-a-chip models to dissect biofilm heterogeneity; (3) dual-omics approaches to map host–pathogen signaling crosstalk; and (4) environmental modulators-such as iron availability and shear stress-that fine-tune virulence expression. Such multidisciplinary efforts will underpin the development of next-generation anti-virulence therapies, ultimately improving prevention and treatment of *P. aeruginosa* infections and safeguarding public health.

## 1 Introduction

*Pseudomonas aeruginosa* is a highly adaptable and virulent opportunistic pathogen that thrives in both natural and hospital environments (Burrows, 2018). It is capable of causing infections ranging from localized wound colonization to life-threatening systemic diseases such as sepsis, with particularly severe outcomes in immunocompromised patients—most notably those with extensive burns or cystic fibrosis (CF) (Elmassry et al., 2020). The organism’s pathogenic success relies on its elaborate repertoire of virulence factors, QS networks, and biofilm-forming capacity, which act in concert to confer formidable immune-evasion and drug-resistance properties (Ng et al., 2023).

Although numerous virulence determinants—such as exotoxin A (Morgan et al., 2021), elastases (Pier, 2007), and multiple dedicated secretion systems (Nans et al., 2015)—have been individually characterized, recent studies indicate that *P. aeruginosa* pathogenicity is seldom attributable to a single factor (Miranda et al., 2022). Rather, it emerges from the coordinated activity of interlinked molecular modules that integrate QS signaling (McCready et al., 2019), extracellular secretion (Alhadrami et al., 2025), and biofilm development, while simultaneously interacting with host immunity and the surrounding micro-environment. This systems-level interplay endows *P. aeruginosa* with exceptional adaptability and persistence.

The principal aim of this review is to dissect the systemic network underpinning *P. aeruginosa* virulence, with a focus on the central regulatory role of QS circuits and their contributions to immune evasion, antimicrobial resistance, and biofilm maturation (Mauad et al., 2023; Shatti et al., 2022; Bao et al., 2019). We highlight three pressing challenges that currently limit translational progress. Although QS antagonists show promise *in vitro*, their clinical deployment remains elusive, partly because QS circuitry displays pronounced plasticity within host environments and can rewire under therapeutic pressure, undermining efficacy. Adaptive reshaping of QS and associated pathways during infection complicates predictions of treatment outcome, necessitating dynamic, rather than static, models of pathogenesis. Many investigations overlook the cooperative or competitive relationships between *P. aeruginosa* and co-colonizing pathogens—interactions that can amplify or dampen virulence outputs and thus modulate therapeutic success.

By delivering a critical synthesis of current knowledge and redirecting attention from single-factor catalogs toward integrated, system-level mechanisms, this review advocates a research paradigm that couples detailed mapping of QS networks with comprehensive studies of host–pathogen interplay and microbial community dynamics. Such an integrative perspective is essential for rationally designing next-generation interventions—spanning anti-biofilm agents, immune modulators, and evolution-conscious therapies—capable of outmaneuvering *P. aeruginosa*’s extensive adaptive arsenal.

## 2 Virulence factors

*Pseudomonas aeruginosa*’s formidable pathogenic capacity is primarily driven by its complex and functionally diverse virulence factor system. These factors play a pivotal role in the infection process by disrupting host defenses, modulating immune responses, and enhancing both colonization and invasive capabilities (Liao et al., 2022). Based on their functional characteristics and mechanisms of action, *P. aeruginosa* virulence factors can be broadly categorized into two main groups: cell-associated factors and extracellular factors (Mauad et al., 2023).

Among the cell-associated factors, flagella and Type IV pili are crucial for bacterial adhesion and motility, serving as essential structural components for initiating infection (Hendrix et al., 2024). Lipopolysaccharides (LPS), on the other hand, not only provoke potent immune responses but also protect the bacteria from host defenses, significantly contributing to the development of bacteremia and sepsis (McCarthy et al., 2017).

Extracellular factors comprise a variety of potent virulence factors and secretion systems. Exotoxin A, for example, inhibits host protein synthesis, inducing cell death (Shadman et al., 2021). Exotoxin S is delivered into host cells via the T3SS, disrupting cellular signaling and promoting bacterial survival within host cells (Dortet et al., 2018). Elastase directly degrades host tissue components, undermining physical barriers to infection (Sun et al., 2020). Additionally, pyocyanin and other pigment metabolites, which exhibit antioxidant properties, enable the bacteria to thrive in oxidative environments (Raoust et al., 2009). The siderophore systems, including pyoverdine and phenazines, enhance virulence expression and biofilm formation by competing for iron resources from the host (Kim et al., 2018).

These virulence factors do not operate in isolation but rather interact in a synergistic manner to augment the pathogenic potential of the bacterium. For instance, T3SS not only facilitates the transmembrane delivery of effector proteins but is also closely linked to bacterial virulence phenotypes and clinical outcomes (Li et al., 2022; Li et al., 2022). Studies have shown that strains carrying the *exoU* gene, which encodes a highly toxic phospholipase, exhibit heightened pathogenicity and are frequently associated with high-virulence clonal types, such as ST235 (Song et al., 2023).

Epidemiological studies indicate that *P. aeruginosa* is one of the primary opportunistic pathogens responsible for nosocomial infections, with a detection rate ranging from 4.7 to 8.9% in bloodstream infections (Mauad et al., 2023). The mortality rate for bacteremia caused by multidrug-resistant strains can reach as high as 20–40% (Herrera et al., 2021; Jarlier et al., 2019; Hickson et al., 2024; Sendra et al., 2024). Furthermore, certain serotypes, such as O1 and O11, and high-risk clonal strains, such as ST235, are closely associated with poorer infection outcomes (Papa-Ezdra et al., 2024; Recio et al., 2018). However, there remains ongoing debate as to whether virulence factors can independently serve as predictors of mortality in bloodstream infections.

### 2.1 Secretion systems

The T3SS is a critical virulence mechanism in *P. aeruginosa*, structurally resembling a “molecular syringe” composed of about 20 proteins that span the bacterial membranes and extend to the host cell membrane (Nans et al., 2015). During infection, T3SS injects multiple effector proteins directly into the host cell cytoplasm, disrupting cellular functions and promoting bacterial colonization and pathogenicity (Wagner et al., 2018). This system is key in acute infections like pneumonia and gastroenteritis, with active T3SS strains showing higher toxicity and drug resistance (Marshall et al., 2013).

The main T3SS effectors in *P. aeruginosa* include ExoS, ExoT, ExoU, and ExoY (Zhu et al., 2023). ExoS and ExoT have GTPase and ADP-ribosyltransferase activities, with ExoS disrupting host cell cytoskeleton and signaling pathways, and ExoT inhibiting cell migration and adhesion (Armentrout et al., 2021). ExoU, a highly toxic phospholipase, rapidly damages host cell membranes, inducing apoptosis and correlating with severe infections (Foulkes et al., 2022). ExoY increases cellular adenosine AMP levels, further disrupting host functions (Armentrout et al., 2021). High-risk clonal strains like ST235, carrying the *exoU* gene, are linked to severe patient conditions, making T3SS a critical virulence biomarker (Song et al., 2023).

In contrast, the Type VI Secretion System (T6SS) is a complex nanoscale “injection device, “similar to bacteriophage tail spikes (Navarro-Monserrat and Taylor, 2023). It delivers toxin proteins into neighboring cells. *P. aeruginosa* encodes three T6SS subtypes: H1-T6SS, H2-T6SS, and H3-T6SS (Robinson et al., 2023). H1-T6SS targets other bacteria, releasing cell wall-degrading enzymes to eliminate competitors, while H2-T6SS and H3-T6SS regulate host interactions and infection processes (Robinson et al., 2023). T6SS enhances bacterial competitiveness, immune evasion, and biofilm stability, contributing to chronic infections and drug resistance, making it a promising target for new anti-infective therapies (Wang et al., 2021; Liao et al., 2022).

### 2.2 Exotoxins

Exotoxin A (ExoA), encoded by the *toxA* gene, is a key virulence factor in *P. aeruginosa*. This 638-amino-acid protein (66 kDa) has specific ADP-ribosyltransferase activity, targeting eukaryotic elongation factor 2 (*eEF-2*) to inhibit protein synthesis, leading to cellular dysfunction, apoptosis, or necrosis (Morgan et al., 2023; Morgan et al., 2021). ExoA is central to *P. aeruginosa*’s pathogenicity (Gholami et al., 2023).

Exotoxin S (ExoS) is transported into host cells via T3SS, containing an ADP-ribosyltransferase domain crucial for intracellular survival (Pinto et al., 2016). ExoS inhibits endocytic vesicle acidification in epithelial cells, helping bacteria evade lysosome-autophagy and intracellular killing (Chu et al., 2024). It also creates a favorable intracellular environment, promoting bacterial survival and enhancing chronic and systemic infection capabilities (Kroken et al., 2022).

### 2.3 Lipopolysaccharides (LPS)

Lipopolysaccharides, a complex and variable component of *P. aeruginosa*’s outer membrane, consists of lipid A, core polysaccharide, and O-antigen (Pier, 2007). Its structural diversity enables antigenic variation, helping bacteria evade the host immune system. LPS forms a physical barrier, enhancing resistance to environmental stress and playing a key role in host-bacteria interactions (Bertani and Ruiz, 2018).

As a pathogen-associated molecular pattern (PAMP), LPS is recognized by the host’s innate immune system via the TLR4 pathway, triggering the production of pro-inflammatory cytokines like TNF-*α*, IL-1, IL-6, and IFN-*γ*, which can cause tissue damage in pulmonary infections (Pier, 2007; Kayagaki et al., 2013). LPS modifications, such as lipid A phosphorylation or acylation, reduce sensitivity to antimicrobial peptides and polymyxin antibiotics (Sciuto et al., 2019). LPS also contributes to outer membrane vesicle (OMV) formation, aiding virulence factor transport, cell communication, and biofilm formation, thereby supporting chronic infections (Augustyniak et al., 2022).

### 2.4 Other virulence factors

In addition to LPS, other structural virulence factors of *P. aeruginosa* also contribute significantly to its pathogenicity. Flagella, as key structures for bacterial motility, not only facilitate swimming in liquid environments and gliding on solid surfaces but also play a crucial role in the early stages of infection by promoting colonization and adhesion (Barken et al., 2008). The flagellum consists of a filament, hook, and basal body, typically forming a monopolar single structure. This structure is recognized by the host’s innate immune system through Toll-like receptor 5 (TLR5), triggering a strong pro-inflammatory response (Kreutzberger et al., 2020). However, to evade continuous immune surveillance, *P. aeruginosa* can downregulate or even shut down flagella expression during infection, exhibiting “stage-specific antigenic variation.” Furthermore, flagella are involved in the early stages of biofilm formation, providing favorable conditions for chronic infection (Ramzan et al., 2022).

Type IV pili are filamentous structures that dynamically extend and retract, mediating “twitching” motility, adhesion, bacterial interactions, and biofilm maturation. They primarily mediate “twitching” motility, adhesion, bacterial interactions, and biofilm maturation (Beaussart et al., 2014). Type IV pili play a critical role in adhering to host cell surfaces, sensing host signals, and regulating virulence factor expression. Strains lacking pili often exhibit reduced adhesion, impaired biofilm formation, and significantly decreased infection efficiency (Barken et al., 2008). Studies suggest that this structure is not only essential for bacterial motility but also serves as an integrated platform for signal transduction and functional output, determining the bacterium’s ability to establish infections in the host (Hendrix et al., 2024).

Additionally, outer membrane vesicles (OMVs), secreted by *P. aeruginosa* throughout its life cycle, have various roles in virulence expression and dissemination (Augustyniak et al., 2022). OMVs are nanoscale vesicles that bud from the outer membrane, containing LPS, proteins, and other virulence molecules, such as Exotoxin A and elastase. These vesicles can deliver virulence factors to host cells, enhancing bacterial damage, and facilitate the spread of drug resistance genes and signaling molecules between bacteria, thereby promoting QS regulation (Qin et al., 2022). OMVs also contribute to biofilm formation and stabilization, showing higher secretion activity in chronic infection environments. As such, they represent a key strategy for *P. aeruginosa* to adapt to host immune defenses and antibiotic pressure (Chen et al., 2024).

It is worth noting that *P. aeruginosa* can also utilize its T3SS and T6SS to survive and proliferate within host cells. The T3SS can directly inject effector proteins into the host cell cytoplasm, disrupting cell functions and inhibiting the host’s immune response, thereby creating favorable conditions for intracellular bacterial survival. The T6SS can deliver toxin proteins to neighboring host cells, further expanding the infection. This ability to grow within host cells is an important virulence strategy of *P. aeruginosa*, allowing it to evade host immune surveillance and persist within the host.

*P. aeruginosa* is a highly adaptable opportunistic pathogen that exhibits formidable pathogenicity through its complex virulence factor system. It has garnered significant attention due to its intrinsic resistance to antibiotics and its ability to cause a wide range of infections, particularly in immunocompromised individuals. These virulence factors disrupt host defenses, modulate immune responses, and enhance bacterial colonization and invasiveness during infection. The pathogenicity of *P. aeruginosa* is primarily mediated by a diverse array of virulence factors, which facilitate evasion of host defenses, modulation of immune responses, and enhancement of colonization and invasion capabilities. Research indicates that certain high-virulence clonal types, such as ST235, are closely associated with severe clinical outcomes. However, whether virulence factors can serve as independent predictors of mortality remains controversial. Despite extensive research into these virulence determinants, it remains unclear whether their expression can be reliably used to predict clinical outcomes, such as mortality in bloodstream infections.

In this review, we critically assess the current understanding of *P. aeruginosa* virulence factors by categorizing them according to their functional characteristics into cell-associated and extracellular factors, and evaluating their respective roles in the pathogenesis of infection. Although certain highly virulent clonal lineages, such as ST235, have been associated with severe infections, it is still debated whether these factors can serve as independent predictors of adverse clinical outcomes.

Furthermore, we explore the secretion systems of *P. aeruginosa*, particularly the T3SS and the T6SS, and examine their interactions with other virulence determinants. While these systems are generally considered central drivers of pathogenicity, the interplay and potential synergistic effects among different virulence mechanisms remain underexplored.

Therefore, the aim of this review is not only to summarize the various pathogenic mechanisms employed by *P. aeruginosa* but also to critically evaluate their potential as biomarkers for predicting infection severity and treatment outcomes.

## 3 Quorum sensing system (quorum sensing, QS)

### 3.1 Composition of the QS system

The QS network of *P. aeruginosa* comprises three interconnected signaling circuits, forming a multi-tiered regulatory hierarchy. These are: LasI/LasR system, which uses N-3-oxododecanoyl-L-homoserine lactone (3OC12-HSL); RhlI/RhlR system, which relies on N-butanoyl-L-homoserine lactone (C4-HSL); PqsR (MvfR) system, which employs 2-heptyl-3,4-dihydroxyquinoline (PQS) and its biosynthetic precursor 2-heptyl-4-quinolone (HHQ) (Pier, 2007).

In the Las circuit, LasI synthesizes 3OC12-HSL, which is actively secreted into the extracellular milieu. Once the cell density rises above a critical threshold, accumulated 3OC12-HSL diffuses back into the cell and binds LasR. This ligand–receptor interaction induces a conformational shift in LasR, triggering transcription of numerous genes responsible for virulence factor production and biofilm development (Wargo and Hogan, 2007). The Rhl module displays distinct diffusion dynamics: C4-HSL, produced by RhlI, freely crosses the cell membrane due to its smaller size and lipophilicity. Upon reaching its activation threshold, extracellular C4-HSL re-enters the cell to bind RhlR, thereby regulating downstream targets such as rhamnolipid synthase and other factors critical for motility and biofilm architecture (Miranda et al., 2022).

Central to this tripartite network is the PqsR system. HHQ and PQS levels are reciprocally modulated by the Las and Rhl circuits, while PQS also enhances Las- and Rhl-dependent signaling via a positive feedback loop. This “Las-Rhl-PQS” regulatory triangle orchestrates key processes-ranging from exotoxin secretion to biofilm maturation and antibiotic tolerance—through precise spatiotemporal control (Wilder et al., 2011). Such layered signal integration allows *P. aeruginosa* to fine-tune communal behaviors in response to fluctuating environmental cues.

### 3.2 Regulatory mechanisms of the QS system

**Figure 1:**
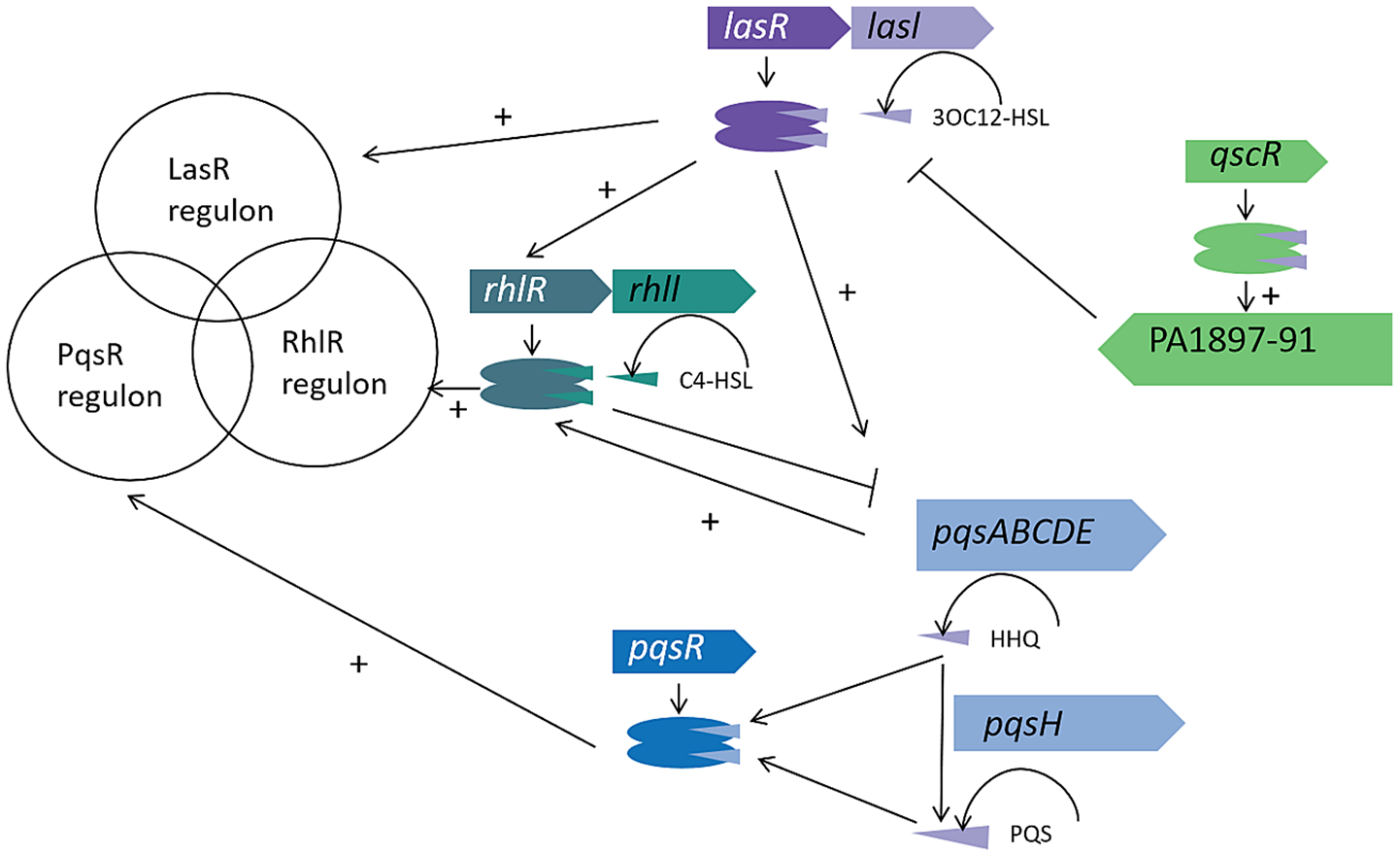
The *Pseudomonas aeruginosa* QS circuitry. The Las, Rhl, and PQS systems in *P. aeruginosa* collectively regulate hundreds of genes in response to increasing cell density. Some virulence factors, such as LasB elastase and phenazine biosynthesis, are controlled by multiple QS systems, while others, like rhamnolipids, are strictly regulated by a single system, in this case, the Rhl system. The QscR regulator binds to the 3OC12-HSL signal produced by LasI and activates the linked PA1897–91 operon. Unlike the AHL-based Las and Rhl systems, the PQS system receptor PqsR responds to both the low-affinity signal HHQ and the high-affinity signal PQS, with HHQ being converted to PQS by the product of the *pqsH* gene. These regulatory systems interact with each other as indicated by the connecting lines.

The three QS systems of *P. aeruginosa* work in concert to form a cascade regulatory network, jointly coordinating the expression regulation of over 400 target genes (Figure 1) (Pier, 2007). Notably, about 20% of these controlled genes are directly involved in the biosynthesis of bacterial virulence factors. This regulatory system dynamically adjusts four core physiological modules through a population density sensing mechanism: (1) the formation and maturation of the three-dimensional structure of biofilms; (2) the transmembrane transport efficiency of multidrug efflux pump systems; (3) the release of effectors of the Type VI Secretion System; (4) the motility and swarming movement mediated by flagella (Hemmati et al., 2024). It is particularly important to note that the system also coordinates the spatiotemporal control of key virulence pathways such as alginate biosynthesis, elastase secretion, pyocyanin production, and iron chelation (Venturi, 2006).

At the molecular regulatory level, each QS system follows the “ligand-receptor” activation paradigm: when specific signal molecules (3OC12-HSL, C4-HSL, PQS/HHQ) bind to their homologous receptors (LasR, RhlR, PqsR), they trigger conformational rearrangements of the receptor proteins, thereby acting as molecular switches to activate target gene transcription (Pier, 2007). This interaction mechanism forms a self-reinforcing signal amplification loop – the activated receptor not only upregulates the expression of signal molecule synthase genes (such as *lasI, rhlI, pqsABCDE*) but also activates secondary regulatory factors (such as *rsaL, qscR*) through a cascade reaction, ultimately driving multidimensional functional modules including virulence effector factors (*exoS*, *exoU*), group behavior regulatory elements (*rhlAB, pelA*), and antibiotic resistance genes (*mexAB-oprM*) (Miranda et al., 2022).

This multi-level signal integration system endows *P. aeruginosa* with unique ecological adaptability: when the population density reaches a critical threshold, the QS network synchronizes gene expression, achieving a strategic shift from individual bacterial behavior to a group pathogenic mode, significantly enhancing its colonization ability and immune evasion efficiency within host tissues.

### 3.3 Relationship between the QS system and bacterial drug resistance

The relationship between the QS system and bacterial antibiotic resistance is intricate and closely intertwined. On one hand, the QS system can regulate the expression of multidrug efflux pump genes, thereby contributing to the development of MDR (Sikdar and Elias, 2022). Multidrug efflux pumps actively extrude antibiotics that have penetrated bacterial cells, thereby lowering their intracellular concentrations and weakening the efficacy of the antibiotics. For example, studies have shown that the expression of specific multidrug efflux pump genes in *P. aeruginosa* is directly regulated by the QS system. Activation of the QS system leads to upregulation of these efflux pump genes, consequently enhancing bacterial resistance to multiple antibiotics (Alcalde-Rico et al., 2018).

On the other hand, alterations in the expression of multidrug efflux pumps can also modulate the QS system itself. Certain efflux pumps are capable of expelling QS signaling molecules from bacterial cells, thereby reducing the extracellular concentrations of these molecules and impairing QS-mediated signal transduction. For instance, overexpression of the MexCD-OprJ and MexEF-OprJ efflux pumps has been shown to expel compounds such as HHQ and kynurenine, leading to the suppression of QS responses in *P. aeruginosa*, a decrease in intracellular PQS levels, and subsequent reduction in the production of QS-regulated virulence factors (Zaborskyte et al., 2017). Moreover, efflux pumps such as MexAB-OprM can extrude acyl-homoserine lactones like 3OC12-HSL, further disrupting normal QS signaling (Wargo and Hogan, 2007).

In clinical settings, infections caused by MDR *P. aeruginosa* present substantial therapeutic challenges. Notably, MDR strains of *P. aeruginosa* are more prone to mutations in the lasR gene, which further compromise QS system functionality and impact bacterial virulence and resistance profiles (Alhadrami et al., 2025). In light of these findings, therapeutic approaches that concurrently target the QS system and efflux pumps emerge as a highly promising strategy for combating multidrug-resistant *P. aeruginosa* infections. By concurrently inhibiting QS signaling and efflux pump activity, the bactericidal efficacy of antibiotics can be significantly enhanced.

The QS system of *P. aeruginosa* is a highly sophisticated regulatory network that orchestrates the expression of over 400 genes via three interconnected circuits—LasI/LasR, RhlI/RhlR, and PqsR. These circuits collectively play pivotal roles in controlling virulence-factor production, biofilm development, multidrug efflux pump efficiency, type VI secretion system activity, and motility. The QS system also promotes multidrug resistance by regulating efflux pump genes. However, overexpression of these pumps can disrupt QS signaling, reducing virulence-factor production. Despite a substantial body of research elucidating key aspects of the QS system, critical knowledge gaps remain, and its full impact on *P. aeruginosa* pathogenicity and drug resistance is not yet completely understood. In this review, we provide a critical appraisal of the molecular mechanisms underpinning QS signaling and its roles in modulating virulence determinants, biofilm formation, and multidrug resistance.

Despite extensive investigation of QS and its well-established contribution to coordinated virulence expression, controversy persists regarding its role in antibiotic-resistance mechanisms in MDR *P. aeruginosa*. The relationship between QS signaling and efflux-pump activity is particularly complex: while QS can up-regulate the transcription of multidrug efflux-pump genes, overexpression of these pumps can, in turn, dampen QS signal transduction and reduce virulence-factor production. This feedback loop has significant implications for the development of effective treatment strategies. However, it remains debated whether targeting the QS network alone can overcome resistance, or whether a more integrated approach—simultaneously inhibiting QS signals and efflux-pump function—will confer greater clinical benefit. Targeting both the QS system and efflux pumps in combination therapies may enhance antibiotic efficacy and offer new strategies for treating multidrug-resistant *P. aeruginosa* infections.

This review critically synthesizes current evidence on how QS influences *P. aeruginosa* pathogenicity and antibiotic resistance, emphasizing the need for more comprehensive studies to resolve the intricate interplay between QS signaling and resistance mechanisms. We contend that, although QS inhibition shows promise as a therapeutic strategy, further exploration of its utility in combination regimens—and a deeper understanding of its broader significance in treating MDR *P. aeruginosa* infections—are urgently required.

## 4 Biofilm formation

The biofilm of *P. aeruginosa* serves as a central regulatory hub for drug resistance and virulence expression, fundamentally reshaping bacterial survival strategies through complex structural and functional networks. The biofilm consists of a three-dimensional matrix formed by extracellular polymeric substances (EPS) secreted by the bacteria. This matrix not only acts as a physical barrier that limits antibiotic penetration but also creates a unique metabolic microenvironment that reduces bacterial susceptibility to antimicrobial agents (Shatti et al., 2022). Within the biofilm, bacterial cells display diverse metabolic statuses, with some cells transitioning into slow-growing or dormant states. This transition markedly reduces antibiotic uptake and metabolic activity, ultimately leading to phenotypic drug resistance (Schiessl et al., 2019).

Furthermore, the high cell density within the biofilm environment regulates the expression of virulence factors through the QS system. For example, QS positively regulates the secretion of T3SS effectors, such as ExoU, enhancing host cell toxicity (Olaniran et al., 2023). In addition, QS promotes biofilm maturation and structural stability by controlling the synthesis of key EPS components, including alginate, thereby strengthening bacterial colonization capabilities (Eltayb et al., 2022).

The biofilm microenvironment also markedly facilitates the horizontal transfer of drug resistance genes. Through vesicle-mediated transport and plasmid conjugation, the dissemination of multidrug efflux pump genes, such as MexAB-OprM, is accelerated (Wargo and Hogan, 2007). These efflux pumps, whose expression is upregulated under QS regulation, further decrease intracellular antibiotic concentrations, enhancing bacterial resistance.

Biofilm-associated virulence is also evident in the bacteria’s enhanced ability to evade host immune responses. EPS components can adsorb immune cell surface receptors, inhibit phagocytosis, and trigger excessive inflammatory responses, ultimately leading to tissue damage (Wan et al., 2024). Notably, biofilm formation is closely associated with the hypervirulent phenotypes of specific clonal strains, such as ST235, which frequently harbor additional virulence islands and drug resistance determinants (Doumith et al., 2022).

In summary, through a combination of physical protection, metabolic adaptation, horizontal gene transfer, and virulence regulation, biofilm formation constitutes a core factor in the persistence and recalcitrance of *P. aeruginosa* infections, providing a theoretical foundation for the development of novel anti-biofilm therapeutic strategies.

### 4.1 Structure and function of biofilms

The biofilm formed by *P. aeruginosa* is a highly organized bacterial community in which cells are embedded within a self-produced EPS matrix. This matrix primarily consists of polysaccharides, extracellular DNA (eDNA), proteins, lipids, and other macromolecules. Together, these components account for more than 90% of the total biofilm biomass and are intricately interwoven to construct the biofilm’s complex three-dimensional architecture (Shatti et al., 2022).

Biofilm development is a dynamic and highly ordered process that typically occurs in six stages. Initially, planktonic bacteria encounter a surface through random movement and begin to adhere via weak interactions. This reversible adhesion subsequently evolves into irreversible attachment as the bacteria proliferate. Subsequently, bacterial cells form microcolonies that mature into structured biofilms, often exhibiting characteristic mushroom-like shapes. During maturation, channels and voids develop within the biofilm to facilitate material exchange and nutrient flow. In the final stage, a subset of cells disperses from the biofilm, reverting to the planktonic state to colonize new niches in the environment (Rasamiravaka et al., 2015).

Biofilms play a critical role in the pathogenicity of *P. aeruginosa*. They provide a stable and protective niche, shielding bacterial cells from host immune responses and limiting antibiotic penetration. The EPS matrix serves as a physical and chemical barrier by impeding the diffusion of antibiotics and by adsorbing or inactivating certain antimicrobial agents (Shatti et al., 2022). In addition, bacteria within the biofilm benefit from cooperative interactions, including the sharing of nutrients, enzymes, and cytoplasmic components, which enhance community survival and resilience. Furthermore, biofilms facilitate bacterial adhesion to both biotic and abiotic surfaces, thereby promoting persistent colonization, especially on host tissues, and contributing to the establishment and chronicity of infections (Schiessl et al., 2019).

### 4.2 Biofilms and bacterial drug resistance

Biofilm-specific antibiotic resistance is a hallmark of *P. aeruginosa* biofilms, characterized by multiple synergistic mechanisms that collectively confer high-level tolerance to antimicrobial agents.

At the gene expression level, biofilm formation leads to significant alterations in the transcriptional landscape of *P. aeruginosa*, directly influencing antibiotic susceptibility. For instance, studies have demonstrated that the expression levels of genes such as *ndvB* and *tssC1* are markedly modulated during biofilm development, and these changes are closely associated with biofilm-specific resistance. These genes may be involved in modifying biofilm architecture, bacterial metabolic states, or antibiotic target accessibility, thereby enhancing bacterial survival under antibiotic stress (Hall et al., 2018).

The extracellular matrix components, particularly polysaccharides such as alginate, Psl, and Pel, play essential roles in structural integrity and drug resistance within the biofilm. Alginate, a mucoid polymer made up of guluronic and mannuronic acids, enhances the viscoelasticity and density of the biofilm matrix, thereby hindering the penetration of antibiotics (Dickerhof et al., 2019). Psl, rich in mannose, glucose, and rhamnose, mediates intercellular interactions and surface adhesion. Pel, a cationic polysaccharide containing *N*-acetylgalactosamine and *N*-acetylglucosamine, facilitates cell–cell and surface adhesion, DNA cross-linking, and has been shown to protect against aminoglycoside antibiotics, enabling bacterial persistence even in high-concentration drug environments (Fleming et al., 2022).

Moreover, bacteria within biofilms often enter a metabolically quiescent or dormant state, distinct from their planktonic counterparts. These cells exhibit reduced metabolic activity and lower rates of antibiotic uptake, rendering them more tolerant to antimicrobial agents (Qi et al., 2022). In addition, QS and intercellular signaling within the biofilm microenvironment modulate gene expression patterns, further promoting antibiotic resistance by coordinating stress responses and biofilm-specific adaptations.

### 4.3 Regulatory mechanisms of biofilm formation

The QS system plays a central regulatory role in the formation of biofilms in *P. aeruginosa*. Studies have shown that biofilms formed by QS-deficient mutants are usually thinner and less developed, and are more sensitive to antibiotic treatment and clearance by the host immune system (Olaniran et al., 2023). This clearly underscores the crucial role of the QS system in the normal development and functional maintenance of biofilms.

During the formation of biofilms, the QS system regulates the expression of a series of genes, affecting various stages of biofilm development. For example, the QS system can positively regulate genes involved in the maturation and persistence of biofilms, promoting bacterial adhesion, EPS synthesis, and the stability of the biofilm structure (Eltayb et al., 2022). In addition to the QS system, other regulatory factors such as cyclic di-GMP (c-di-GMP) also play a key role in biofilm formation. C-di-GMP can stimulate the synthesis of biofilm matrix components, especially the production of polysaccharides such as Psl, Pel, and alginate. When the intracellular level of c-di-GMP increases, it promotes the synthesis and secretion of these polysaccharides, thereby promoting the formation and development of biofilms (Yan et al., 2010).

Different strains of *P. aeruginosa* have differences in the signaling pathways controlling the initiation of biofilm formation. For example, the PAO1 strain promotes bacterial adhesion to surfaces and matrix secretion through the Wsp system-mediated increase in c-di-GMP production, initiating the biofilm formation process (Ravichandran et al., 2015). In contrast, the PA14 strain mainly controls surface typing through the Pil-Chp system, affecting the initiation of biofilm formation (Lee et al., 2020). These different regulatory mechanisms indicate that *P. aeruginosa* has evolved a variety of biofilm formation regulatory strategies to adapt to different environments, ensuring successful biofilm formation and enhanced survival and pathogenic capabilities in various environments.

Biofilm formation is a fundamental feature of *P. aeruginosa* pathogenicity and lies at the core of the bacterium’s persistence within host tissues. *P. aeruginosa* biofilms are central to drug resistance and virulence expression. The biofilm matrix, composed of EPS secreted by the bacterium, together with densely packed cells, limits antibiotic penetration and creates a unique metabolic microenvironment. High cell density within biofilms regulates virulence factor expression via QS, enhancing host cell toxicity and biofilm stability. Moreover, the biofilm micro-environment enhances evasion of host immune defences and modulates the expression of numerous virulence determinants. Biofilms also facilitate the horizontal transfer of drug resistance genes, promoting antibiotic resistance. Additionally, biofilm components inhibit phagocytosis and trigger inflammation, aiding bacterial evasion of host immune responses.

A critical issue concerns whether current anti-biofilm strategies adequately address the multifaceted nature of biofilm-associated resistance. Evidence indicates that biofilm-embedded bacteria frequently adopt a dormant phenotype, rendering them less susceptible to conventional antibiotics; yet the impact of these dormant states on therapeutic outcomes, and the conditions under which reactivation may occur, remain unclear. Furthermore, biofilm components such as EPS and QS regulatory factors promote bacterial survival under antibiotic pressure, but their interactions with host immune responses have not been fully characterized. Targeting biofilms is crucial for developing effective anti-infective strategies.

Although the role of biofilms in chronic infection is well documented, the extent to which biofilm dynamics influence the efficacy of therapeutic strategies against *P. aeruginosa* remains incompletely understood. This review critically examines the current understanding of *P. aeruginosa* biofilm development, with particular emphasis on its contribution to antimicrobial resistance and virulence-factor regulation. While the protective nature of biofilms is widely recognized, the intricate processes governing biofilm maturation and their interplay with QS signaling have not been fully elucidated. In addition, debate persists over whether biofilm-specific resistance arises predominantly from metabolic adaptations within sessile cells or from the structural barrier imposed by the EPS matrix. Although biofilms are generally considered the principal impediment to successful treatment of *P. aeruginosa* infections, there is still no consensus on how to target biofilms effectively without disrupting beneficial host–microbe interactions.

## 5 Intracellular survival and growth of *P. aeruginosa*

*Pseudomonas aeruginosa* has developed complex strategies for intracellular survival and proliferation, which significantly enhance its virulence and contribute to the persistence of chronic infections (Rossi et al., 2021). This ability allows the bacterium to evade host immune surveillance and establish long-term infections, particularly in immunocompromised individuals and those with underlying conditions such as CF (Bhagirath et al., 2016).

### 5.1 Mechanisms of intracellular survival

*P. aeruginosa* utilizes its T3SS and T6SS to facilitate intracellular survival (Li et al., 2020). The T3SS injects effector proteins directly into host cells, disrupting cellular functions and inhibiting host immune responses (Wagner et al., 2018). For instance, ExoU, a highly toxic phospholipase, rapidly damages host cell membranes, inducing apoptosis and correlating with severe infections (Dortet et al., 2018). The T6SS, on the other hand, delivers toxin proteins to neighboring host cells, further expanding the infection (Robinson et al., 2023).

### 5.2 Impact on infection dynamics

Intracellular growth of *P. aeruginosa* is particularly significant in chronic infections, where it can form biofilms within host tissues (Rossi et al., 2021). These biofilms provide a protective environment, enhancing bacterial resistance to antibiotics and immune responses. Studies have shown that *P. aeruginosa* can survive within macrophages and epithelial cells, contributing to persistent infections in CF patients (Bhagirath et al., 2016). This intracellular environment enables the bacterium to avoid phagocytosis and other immune defenses, thus posing a significant challenge to conventional therapeutic approaches.

### 5.3 Implications for treatment

The ability of *P. aeruginosa* to grow within host cells complicates treatment strategies, as intracellular bacteria are often protected from antibiotics. Inhibiting intracellular survival mechanisms, particularly the functions of the T3SS and T6SS, emerges as a promising strategy for developing novel therapeutic approaches. Small-molecule inhibitors targeting T3SS have demonstrated significant potential in diminishing bacterial virulence and enhancing treatment efficacy (Gu et al., 2015).

*Pseudomonas aeruginosa* has evolved complex mechanisms to survive and proliferate within host cells, significantly enhancing its virulence and persistence in chronic infections. It utilizes type III and type VI secretion systems to disrupt host cell functions and evade immune responses. The formation of biofilms within host cells further protects it from immune surveillance and antibiotics. These intracellular survival and replication strategies significantly enhance its pathogenic potential and contribute to the persistence of chronic infections, particularly in immunocompromised individuals and patients with underlying conditions such as CF. Although the ability of *P. aeruginosa* to survive within host cells has been well documented, the precise mechanisms underlying this survival and their impact on therapeutic outcomes remain incompletely understood.

This review critically discusses the implications of *P. aeruginosa* intracellular survival for existing treatment strategies. The bacterium’s resistance to conventional antibiotics within host cells underscores the urgent need to develop novel therapeutic approaches. Targeting T3SS and T6SS represents a promising strategy for impairing intracellular survival mechanisms and, consequently, reducing bacterial virulence. However, further research is necessary to determine how best to inhibit these secretion systems without eliciting adverse host responses.

In conclusion, while intracellular survival and biofilm formation are pivotal to the persistence of *P. aeruginosa*, the complexity of these processes presents significant challenges for the design of effective therapies. A deeper understanding of the intricate host–pathogen interactions is essential for the development of new therapeutic strategies capable of overcoming the limitations of current treatments.

## 6 Immune evasion mechanisms of *P. aeruginosa*

The immune evasion mechanisms of *P. aeruginosa*, in conjunction with its virulence and antibiotic resistance strategies, constitute a central factor in the persistent nature of infections caused by this pathogen.

In terms of virulence, the extracellular polysaccharides (EPS) produced by *P. aeruginosa* form a protective mucus layer that interferes with complement activation, inhibits phagocytosis, and induces an inflammatory response (Eltayb et al., 2022). Additionally, the variability of the O-antigen in LPS reduces immune recognition, thereby facilitating long-term bacterial colonization within the host (Uruén et al., 2020). Exotoxin S is another key virulence factor, which disrupts macrophage autophagy and apoptosis via secretion systems, thereby enhancing bacterial invasiveness (Kroken et al., 2022).

Regarding antibiotic resistance, the EPS matrix within biofilms serves not only as a physical barrier to immune cells and antibiotics but also adsorbs antimicrobial agents through the alginate component of the matrix (Shatti et al., 2022). Furthermore, modifications to the LPS molecule alter the permeability of the bacterial membrane, thereby enhancing the bacteria’s resistance to a variety of drugs (Bertani and Ruiz, 2018). High-risk clonal strains, such as ST235, exhibit elevated virulence and drug resistance due to the synergistic effects of immune evasion, biofilm formation, and antibiotic resistance (Doumith et al., 2022).

This interconnected “immune evasion-virulence-drug resistance” mechanism enables *P. aeruginosa* to effectively adapt to both host immune defenses and antibiotic pressures. Targeting key components of this synergy, such as the synthesis of EPS and the modification of LPS, may provide new therapeutic approaches for treating infections caused by this pathogen.

### 6.1 Host immune response

When the host encounters a *P. aeruginosa* infection, it activates a series of complex and finely tuned immune responses. A prominent feature in pulmonary infections is persistent neutrophil infiltration. Neutrophils rapidly chemotax to the infection site, where they attempt to eliminate *P. aeruginosa* through phagocytosis (Dickerhof et al., 2019). However, in certain conditions, such as in CF patients, genetic defects cause alterations in ion transport and mucus properties on the airway surface. These alterations compromise the functionality of neutrophils, thereby impeding their capacity to effectively eliminate bacteria (Rada, 2017). Instead, excessive neutrophil infiltration triggers a chronic inflammatory response, leading to further lung tissue damage (Fleming et al., 2022; Brencic et al., 2009).

Macrophages also play a crucial role in the immune defense. They recognize and phagocytose *P. aeruginosa*, killing the bacteria through mechanisms such as the production of reactive oxygen species (ROS) and reactive nitrogen species (RNS) (Zhu et al., 2020). However, *P. aeruginosa* has evolved strategies to counteract macrophage-mediated clearance. It secretes specific virulence factors to inhibit macrophage phagocytosis or creates a microenvironment within the macrophages that supports its survival, thus evading complete elimination.

In addition, specific subsets of T helper cells are involved in the immune response. Th1 cells secrete interferon-*γ* (IFN-γ), which activates macrophages and enhances their bactericidal capabilities (Lyadova and Panteleev, 2015). Th17 cells orchestrate the recruitment of neutrophils and other immune cells to modulate inflammatory responses. However, in *P. aeruginosa* infections, Th17 cell dysfunction can occur, preventing effective control of the infection and instead exacerbating tissue damage (Wolf et al., 2016).

Host cells recognize pathogen-associated molecular patterns (PAMPs) and damage-associated molecular patterns (DAMPs) of *P. aeruginosa* through pattern recognition receptors (PRRs). The expression of PRRs varies between cell types. For example, airway epithelial cells express various Toll-like receptors (TLRs), including TLR2, which recognizes *P. aeruginosa* lipoproteins, and TLR4, which detects its lipopolysaccharide (LPS) (John et al., 2010). The assembly of inflammasomes also plays a critical role in the immune response to *P. aeruginosa*. The NLRP3 inflammasome can be activated by bacterial components, triggering the maturation and release of inflammatory cytokines such as interleukin-1β (IL-1β) and initiating inflammatory responses (Broz et al., 2010). The NLRC4 inflammasome, involved in recognizing flagellin, also regulates immune responses during infection (Broz et al., 2010). However, in CF patients, defects in PRR signaling pathways facilitate *P. aeruginosa* colonization of the lungs, setting the stage for chronic infection (Bhagirath et al., 2016).

### 6.2 Immune evasion strategies

Throughout its long evolutionary history, *P. aeruginosa* has developed a range of highly sophisticated immune evasion strategies. These mechanisms can be broadly categorized into three main types: inhibition of complement activation, binding of inhibitory complement regulatory proteins, and inactivation of complement components (González-Alsina et al., 2023).

With regard to polysaccharide-related immune evasion, components such as alginate, Psl, and Pel play a pivotal role in the establishment of chronic biofilm infections and immune evasion. These polysaccharides alter the surface characteristics of the bacteria, thereby impeding host immune system recognition and subsequent bacterial clearance (Fleming et al., 2022). For instance, the alginate polysaccharide forms a dense mucus layer that envelops the bacterial surface, preventing complement activation components from accessing antigenic sites, effectively blocking the complement cascade (Dickerhof et al., 2019).

Additionally, the O-antigen of LPS contributes significantly to immune evasion in *P. aeruginosa*. The O-antigen structure is highly variable, and changes in its composition and distribution can reduce the pathogen’s visibility to the host immune system. *P. aeruginosa* can modify the O-antigen structure, preventing immune cell receptors from recognizing the bacterial surface, thus reducing the likelihood of phagocytosis and enhancing the pathogen’s ability to establish chronic infections within the host (Azimi et al., 2021).

Beyond these mechanisms, *P. aeruginosa* can also modulate its virulence through interactions with other pathogens. For example, in CF lung infections, *P. aeruginosa* often coexists with other pathogens such as *Staphylococcus aureus* and fungi (Fischer et al., 2021). The interactions between these pathogens may impact the virulence of *P. aeruginosa*. On one hand, the presence of other pathogens may alter the host’s immune response, thereby providing a more favorable environment for *P. aeruginosa* infection. On the other hand, *P. aeruginosa* may regulate the expression of its virulence factors through competition or cooperation with other pathogens to better adapt to the host environment and enhance its pathogenicity. Therefore, studying the interactions between *P. aeruginosa* and other pathogens is of great significance for a deeper understanding of its virulence mechanisms.

*Pseudomonas aeruginosa* has evolved a range of sophisticated immune evasion strategies that enable it to circumvent host defense mechanisms and establish persistent infections. *P. aeruginosa* employs diverse mechanisms to evade host immune responses, thereby enhancing its virulence and persistence. Its extracellular polysaccharides interfere with complement activation and phagocytosis, while the variability of its LPS O-antigen reduces immune recognition. Additionally, interactions with other pathogens can modulate its virulence. Targeting these immune evasion strategies may offer new therapeutic approaches against *P. aeruginosa* infections.

The host immune response to *P. aeruginosa* is highly complex, involving the coordinated actions of neutrophils, macrophages, T-helper cells, and PRRs, all of which play critical roles during the course of infection. Nevertheless, despite the robustness of the host’s defense systems, *P. aeruginosa* is able to persist, particularly in chronic infections such as those observed in CF patients, by deploying multiple mechanisms that evade immune surveillance and clearance.

A key issue lies in the role of exopolysaccharides—such as alginate, Psl, and Pel—in inhibiting complement activation and phagocytosis. These polysaccharides form a physical barrier on the bacterial surface, obstructing immune recognition and clearance. However, it remains unclear whether targeting these components can effectively enhance immune clearance without disrupting innate immune function or inducing adverse effects. Additionally, the structural variability of the LPS O-antigen complicates immune recognition, raising questions about the long-term efficacy of therapeutic strategies targeting LPS modifications.

Another critical aspect is the interaction of *P. aeruginosa* with other pathogens, particularly in polymicrobial infections such as those seen in the CF lung. These interactions may modulate *P. aeruginosa* virulence and alter the host immune response. Although studies have identified potential synergistic or antagonistic relationships among co-infecting pathogens, the implications of such interactions for treatment strategies remain poorly understood. The ability of *P. aeruginosa* to adapt to the host environment through these interactions suggests that a multifaceted therapeutic approach—one that simultaneously targets immune evasion mechanisms and inter-pathogen dynamics—may be required.

This review aims to critically evaluate the immune evasion strategies employed by *P. aeruginosa* during infection, with a particular focus on the roles of exopolysaccharides, LPS modifications, and pathogen–host interactions in modulating immune responses. While the importance of these mechanisms in immune evasion has been well documented, significant gaps remain in our understanding of the precise molecular pathways involved. In particular, how these mechanisms evolve over time to enhance *P. aeruginosa*’s resistance to host immunity warrants further investigation.

## 7 Virulence regulation network of *P. aeruginosa*

### 7.1 Global regulatory factors

**Figure 2:**
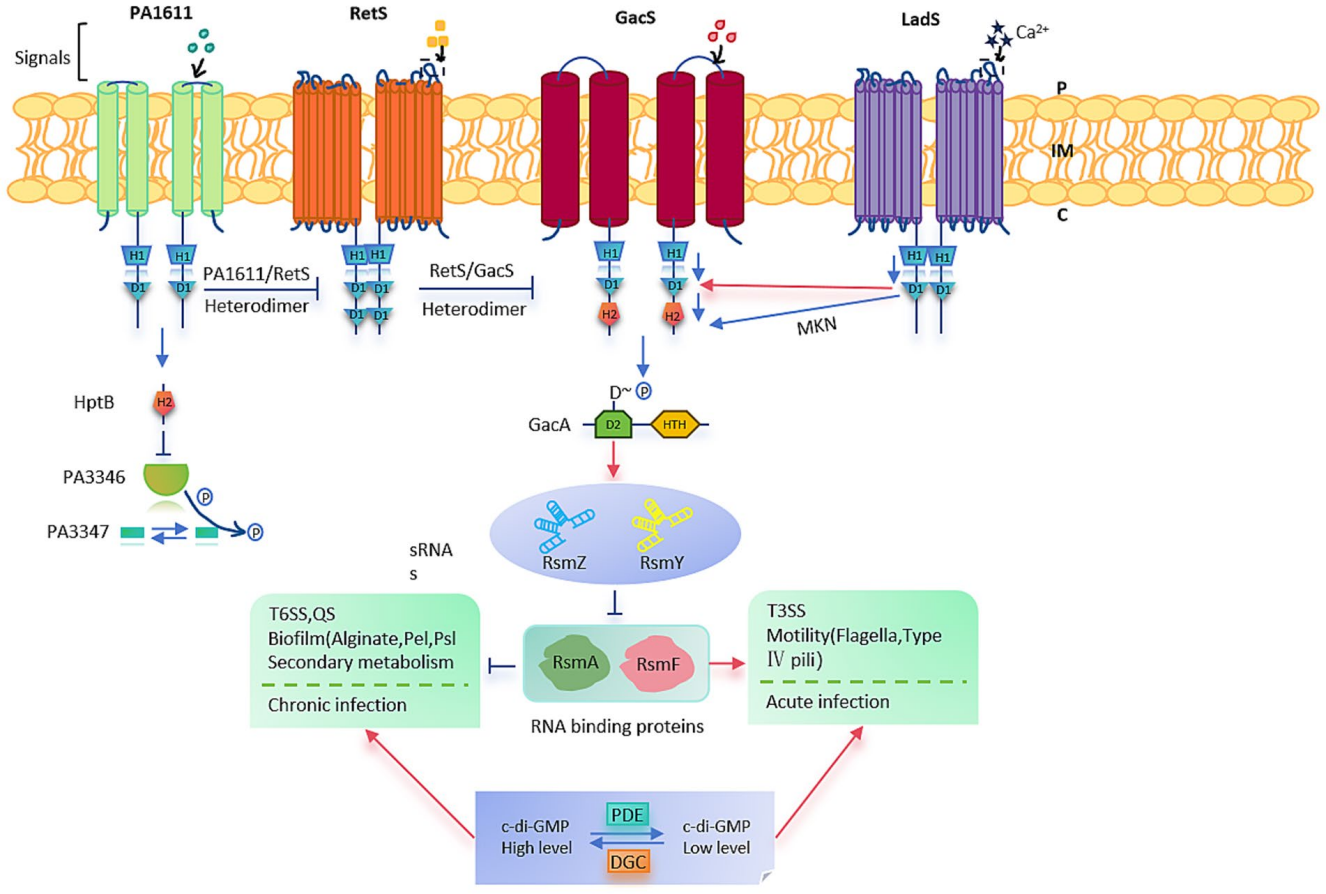
*Pseudomonas aeruginosa* PAO1. Summary of the Gac/Rsm signaling cascade pathway and regulatory phenotypes in *P. aeruginosa* PAO1.

The two-component system (TCS) is one of the primary signal transduction mechanisms in bacteria, typically consisting of a transmembrane sensor histidine kinase (HK) and a cytoplasmic response regulator (RR). It represents a classical stimulus–response coupling model that allows bacteria to adapt rapidly to environmental changes (Fischer et al., 2021). In *P. aeruginosa*, a non-canonical TCS comprising the sensor histidine kinase GacS (initially named LemA) and the response regulator GacA is widely conserved and plays a pivotal role in bacterial physiology and pathogenesis (Song et al., 2023) (Figure 2).

The GacS-GacA TCS functions via a phosphorylation cascade to regulate numerous bacterial activities. Upon sensing an undefined environmental signal, GacS undergoes autophosphorylation and subsequently transfers the phosphate group to GacA, thereby activating it (Zha et al., 2014). Activated GacA binds specific DNA sequences to modulate the transcription of downstream genes (Song et al., 2023). This signaling cascade is crucial for toggling between acute and chronic infection modes: heightened levels of phosphorylated GacA spur biofilm formation and the expression of genes tied to chronic infection, while diminished phosphorylation boosts the expression of acute virulence genes.

This system primarily functions at the post-transcriptional level through the Gac/Rsm signaling pathway. In addition to direct phosphorylation by GacS, hybrid sensor kinases such as LadS (which responds to Ca^2+^), RetS (responsive to mucin polysaccharides), and PA1611 modulate GacA phosphorylation indirectly. Although the exact signals sensed by GacS and PA1611 remain unclear, these kinases regulate target gene expression via complex signal integration (Reimmann et al., 2005).

Functionally, the GacS-GacA system positively regulates the production of several secondary metabolites, including siderophores, pyocyanin, and hydrogen cyanide, as well as various extracellular enzymes. It is also involved in modulating the T3SS, associated with acute infections, and the T6SS, associated with chronic infections. Moreover, by regulating biofilm formation and motility, this system contributes to antibiotic resistance (Corley et al., 2022). The broader RetS-GacS/GacA signaling network plays a crucial role in the transition between planktonic and sessile lifestyles and exerts antagonistic effects in the regulation of phage susceptibility, equipping *P. aeruginosa* with versatile strategies to adapt to diverse host environments (Song et al., 2023).

### 7.2 Influence of environmental signals

Environmental factors, such as oxygen concentration and iron ion availability, significantly influence the virulence of *P. aeruginosa* (Behzadi et al., 2022). In the lungs of CF patients, a hypoxic or even anaerobic microenvironment gradually develops as chronic infections progress. Studies have shown that under low-oxygen conditions, *P. aeruginosa* activates specific gene expression programs that promote the development of dense and robust biofilms (Schaible et al., 2012). This response is mediated by the production of regulatory factors, such as the FNR (fumarate and nitrate reduction) protein, which regulates the expression of biofilm-associated genes, facilitating bacterial adhesion and EPS synthesis (Barbieri et al., 2014).

Pyocyanin, a key pigment produced by *P. aeruginosa*, is synthesized through an oxygen-dependent pathway (Arora et al., 2018). Under aerobic conditions, *P. aeruginosa* efficiently produces pyocyanin, a compound with strong redox activity that induces oxidative stress in host cells. This disruption of the cellular redox balance alters the function of immune cells, contributing to the persistence of chronic infections (Ernst et al., 2022).

Iron is a vital element for the growth and virulence of *P. aeruginosa*, but excessive intracellular iron is toxic. To preserve iron homeostasis, *P. aeruginosa* has developed a complex regulatory mechanism. In iron-limited environments, the bacterium synthesizes various iron chelators, such as pyoverdine and siderophores, to efficiently scavenge iron from the surroundings (Bao et al., 2019). Interestingly, iron limitation also affects biofilm formation. Studies have shown that under low iron conditions, the iron chelators produced by *P. aeruginosa* interact with biofilm components, disrupting the normal structure and function of the biofilm and inhibiting its formation (McDaniel et al., 2016). Moreover, under anaerobic conditions, *P. aeruginosa* becomes more sensitive to chemical chelators, possibly due to changes in metabolic pathways and cell membrane permeability induced by the anaerobic environment (McDaniel et al., 2016).

*Pseudomonas aeruginosa*’s virulence regulation network is complex, involving multiple global regulatory factors and environmental signals. The GacS-GacA two-component system regulates biofilm formation, secondary metabolite production, and various secretion systems through a phosphorylation cascade, affecting transitions between acute and chronic infection modes. Environmental signals such as oxygen concentration and iron availability significantly impact its virulence. Low-oxygen conditions promote biofilm development, while iron limitation affects biofilm structure and function. These regulatory mechanisms enable *P. aeruginosa* to adapt to diverse host environments and persist in infections.

## 8 Conclusion and future perspectives

The virulence mechanisms of *P. aeruginosa* form a highly complex and multidimensionally intertwined network, encompassing various virulence factors, QS systems, biofilm formation, immune evasion mechanisms, and virulence regulation pathways. These mechanisms coordinate and interact with each other, endowing *P. aeruginosa* with significant pathogenicity and environmental adaptability, enabling it to thrive under diverse and fluctuating environmental conditions.

This article systematically reviews the current research on the virulence mechanisms of *P. aeruginosa*. Studies have shown that *P. aeruginosa* directly interferes with the normal physiological functions of host cells via multiple virulence factors, including Exotoxin A (Morgan et al., 2021), Exotoxin S (Pinto et al., 2016), and the Type III (Nans et al., 2015) and Type VI (Navarro-Monserrat and Taylor, 2023) secretion systems. These factors promote bacterial adhesion, invasion, and the establishment of persistent infections. Additionally, the QS system, functioning as a central regulator of bacterial social behavior, governs the expression of a multitude of genes linked to pathogenicity. These include genes involved in biofilm formation, multidrug efflux pump activity, secretion system functions, and bacterial motility (Pier, 2007). This regulation enables *P. aeruginosa* to survive and enhance its drug resistance in response to various stress conditions.

Biofilm formation not only serves as an effective physical barrier against antibiotics and immune system attacks but also optimizes persistent bacterial colonization and dissemination within the host through its unique structural and metabolic characteristics (Shatti et al., 2022). Moreover, the immune evasion strategies of *P. aeruginosa* allow it to effectively escape host immune surveillance, persist within host tissues, and repeatedly induce chronic infections, thereby complicating treatment efforts (Schiessl et al., 2019).

It is also important to recognize the significant impact of environmental factors on the virulence regulation of *P. aeruginosa*. Under hypoxic conditions, *P. aeruginosa* activates specific virulence factor expression programs that promote biofilm formation, thereby enhancing its survival and drug resistance capabilities (Behzadi et al., 2022). In environments where iron is scarce, the bacterium produces a variety of iron chelators to effectively sequester the limited available iron, which in turn affects the regulation of virulence genes. These environmental adaptations greatly contribute to its pathogenicity (Bao et al., 2019). Furthermore, the intracellular growth of *P. aeruginosa* and its interactions with other pathogens are also important components of its virulence strategies.

In conclusion, the virulence mechanisms of *P. aeruginosa* represent a dynamic and highly regulated system, influenced by a combination of virulence factors, regulatory networks, and environmental conditions. Future research on *P. aeruginosa* should focus on several key areas: developing specific inhibitors to target the QS system and disrupt bacterial communication, thereby reducing virulence (Liu et al., 2024; Liu et al., 2022); conducting in-depth analysis of biofilm formation and regulation to identify new intervention targets and enhance biofilm removal strategies; investigating the interaction mechanisms between *P. aeruginosa* and the host immune system to provide a basis for immune modulation therapies; and exploring how environmental factors influence the virulence regulation network to optimize infection prevention and treatment strategies. Potential solutions encompass the development of small-molecule inhibitors targeting QS systems and the application of synthetic biology to engineer novel inhibitors specifically directed at QS pathways. Promising evidences for natural (Ma et al., 2024; Zhou et al., 2018) and synthetic small-molecule QS inhibitors can be translated in several clinical settings such as CF (Carullo et al., 2023). Additionally, targeting biofilm regulatory elements like c-di-GMP signaling pathways, modulating the host immune response through immunostimulatory agents or vaccines, and manipulating environmental conditions such as oxygen levels and iron availability could provide effective strategies for combating *P. aeruginosa* infections (Llamas and Sánchez-Jiménez, 2022). These advancements are expected to offer new theoretical insights and therapeutic strategies, support the development of new drugs and treatments, and ultimately improve patient outcomes, reduce infection rates, and address the global challenge of antibiotic resistance.
